# How the strengths of Lisp-family languages facilitate building complex and flexible bioinformatics applications

**DOI:** 10.1093/bib/bbw130

**Published:** 2016-12-31

**Authors:** Bohdan B Khomtchouk, Edmund Weitz, Peter D Karp, Claes Wahlestedt

**Affiliations:** Center for Therapeutic Innovation and Department of Psychiatry and Behavioral Sciences, University of Miami Miller School of Medicine, 1120 NW 14th St., Miami, FL, USA

**Keywords:** lisp, software engineering, bioinformatics, computational biology, programming languages

## Abstract

We present a rationale for expanding the presence of the Lisp family of programming languages in bioinformatics and computational biology research. Put simply, Lisp-family languages enable programmers to more quickly write programs that run faster than in other languages. Languages such as Common Lisp, Scheme and Clojure facilitate the creation of powerful and flexible software that is required for complex and rapidly evolving domains like biology. We will point out several important key features that distinguish languages of the Lisp family from other programming languages, and we will explain how these features can aid researchers in becoming more productive and creating better code. We will also show how these features make these languages ideal tools for artificial intelligence and machine learning applications. We will specifically stress the advantages of domain-specific languages (DSLs): languages that are specialized to a particular area, and thus not only facilitate easier research problem formulation, but also aid in the establishment of standards and best programming practices as applied to the specific research field at hand. DSLs are particularly easy to build in Common Lisp, the most comprehensive Lisp dialect, which is commonly referred to as the ‘programmable programming language’. We are convinced that Lisp grants programmers unprecedented power to build increasingly sophisticated artificial intelligence systems that may ultimately transform machine learning and artificial intelligence research in bioinformatics and computational biology.

## Introduction and background

The programming language Lisp is credited for pioneering fundamental computer science concepts that have influenced the development of nearly every modern programming language to date. Concepts such as tree data structures, automatic storage management, dynamic typing, conditionals, exception handling, higher-order functions, recursion and more have all shaped the foundations of today’s software engineering community. The name Lisp derives from ‘List processor’ [1], as linked lists are one of Lisp’s major data structures, and Lisp source code is composed of lists. Lists, which are a generalization of graphs, are extraordinarily well supported by Lisp. As such, programs that analyze sequence data (such as genomics), graph knowledge (such as pathways) and tabular data (such as that handled by R [2]) can be written easily, and can be made to work together naturally in Lisp. As a programming language, Lisp supports many different programming paradigms, each of which can be used exclusively or intermixed with others; this includes functional and procedural programming, object orientation, meta programming and reflection.

But more to the point, we have empirical evidence that Lisp is a more productive general-purpose programming language than the other usual suspects, and that most Lisp programs run faster than their counterparts in other languages. Gat [3] compared the run times, development times and memory usage of 16 programs written by 14 programmers in Lisp, C/C ++ and Java. Development times for the Lisp programs ranged from 2 to 8.5 h, compared with 2 to 25 h for C/C ++ and 4 to 63 h for Java (programmer experience alone does not account for the differences). The Lisp programs were also significantly shorter than the other programs.

And although the execution times of the fastest C/C ++ programs were faster than the fastest Lisp programs, on average, the Lisp programs ran significantly faster than the C/C ++ programs and much faster than the Java programs (mean runtimes were 41 s for Lisp versus 165 s for C/C ++).

## Lisp applications and dialects

In bioinformatics and computational biology, Lisp has successfully been applied to research in systems biology [4, 5], high-performance computing (HPC) [6], database curation [7, 8], drug discovery [9], computational chemistry and nanotechnology [10, 11], network and pathway -omics analysis [12, 13, 14, 15, 16], single-nucleotide polymorphism analysis [17, 18, 19] and RNA structure prediction [20, 21, 22]. In general, the Lisp family of programming languages, which includes Common Lisp, Scheme and Clojure, has powered multiple applications across fields as diverse as [23]: animation and graphics, artificial intelligence (AI), bioinformatics, B2B and e-commerce, data mining, electronic design automation/semiconductor applications, embedded systems, expert systems, finance, intelligent agents, knowledge management, mechanical computer-aided design (CAD), modeling and simulation, natural language, optimization, risk analysis, scheduling, telecommunications and Web authoring.

Programmers often test a language’s mettle by how successfully it has fared in commercial settings, where big money is often on the line. To this end, Lisp has been successfully adopted by commercial vendors such as the Roomba vacuuming robot [24, 25], Viaweb (acquired by Yahoo! Store) [26], ITA Software (acquired by Google Inc. and in use at Orbitz, Bing Travel, United Airlines, US Airways, etc.) [27], Mirai (used to model the Gollum character for the Lord of the Rings movies) [28], Boeing [29], AutoCAD [30], among others. Lisp has also been the driving force behind open source applications like Emacs [31] and Maxima [32], which both have existed for decades and continue to be used worldwide.

Among the Lisp-family languages (LFLs), Common Lisp has been described as the most powerful and accessible modern language for advanced biomedical concept representation and manipulation [33]. For concrete code examples of Common Lisp’s dominance over mainstream programming languages like R and Python, we refer the reader to Sections 4 and 5 of Ross Ihaka’s (creator of the R programming language) seminal paper [34].

Scheme [35] is an elegant and compact version of Common Lisp that supports a minimalistic core language and an excellent suite of language extension tools. However, Scheme has traditionally mainly been used in teaching and computer science research and its implementors have thus prioritized small size, the functional programming paradigm and a certain kind of ‘cleanliness’ over more pragmatic features. As such, Scheme is considered far less popular than Common Lisp for building large-scale applications [24].

The third most common LFL, Clojure [36, 37], is a rising star language in the modern software development community. Clojure specializes in the parallel processing of big data through the Java Virtual Machine (JVM), recently making its debut in bioinformatics and computational biology research [38, 39, 40]. Most recently, Clojure was used to parallelize the processing and analysis of SAM/BAM files [39]. Furthermore, the BioClojure project provides seeds for the bioinformatics community that can be used as building blocks for writing LFL applications. As of now, BioClojure consists of parsers for various kinds of file formats (UniProtXML, Genbank XML, FASTA and FASTQ), as well as wrappers of select data analysis programs (BLAST, SignalP, TMHMM and InterProScan) [39].

As a whole, Lisp continues to develop new offshoots. A relatively recent addition to the family is Julia [41]. Although it is sometimes touted ‘C for scientists’ and caters to a different community because of its syntactical proximity to Python, it is a Lisp at heart and certainly worth watching.

## Rewards and challenges

In general, early adopters of a language framework are better poised to reap the scientific benefits, as they are the first to set out building the critical libraries, ultimately attracting and retaining a growing share of the research and developer community. As library support for bioinformatics tasks in the Lisp family of programming languages (Clojure, Common Lisp and Scheme) is yet in its early stages and on the rise, and there is (as of yet) no officially established bioinformatics Lisp community, there is plenty of opportunity for high-impact work in this direction.

It is well known that the best language to choose from should be the one that is most well suited to the job at hand. Yet, in practice, few programmers may consider a nonmainstream programming language for a project, unless it offers strong, community-tested benefits over its popular contenders for the specific task under study. Often times, the choice comes down to library support: does language X already offer well-written, optimized code to help solve my research problem, as opposed to language Y (or perhaps language Z)? In general, new language adoption boils down to a chicken-and-egg problem: without a large user base, it is difficult to create and maintain large-scale, reproducible tools and libraries. But without these tools and libraries, there can never be a large user base. Hence, a new language must have a big advantage over the existing ones and/or a powerful corporate sponsorship behind it to compete [42]. Most often, a positive feedback loop is generated by repositories of useful libraries attracting users, who, in turn, add more functional libraries, thereby raising a programming language’s popularity, rather than reflecting its theoretical potential.

With mainstream languages like R [2] and Python [43] dominating the bioinformatics and computational biology scene for years, large-scale software development and community support for other less popular language frameworks have waned to relative obscurity. Consequently, languages winning over increasingly growing proportions of a steadily expanding user base have the effect of shaping research paradigms and influencing modern research trends. For example, R programming generally promotes research that frequently leads to the deployment of R packages to Bioconductor [44], which has steadily grown into the largest bioinformatics package ecosystem in the world, whose package count is considerably ahead of BioPython [45], BioClojure [38], BioPerl [46], BioJava [47], BioRuby [48], BioJulia [49] or SCABIO [50]. Given the choice, R programmers interested in deploying large-scale applications are more likely to branch out to releasing Web applications (e.g. Shiny [51]) than to graphical user interface (GUI) binary executables, which are generally more popular with lower-level languages like C/C ++ [52]. As such, language often dictates research direction, output and funding. Questions like ‘who will be able to read my code?’, ‘is it portable?’, ‘does it already have a library for that?’ or ‘can I hire someone?’ are pressing questions, often inexorably shaping the course and productivity of a project. However, despite its popularity, R has been severely criticized for its many shortcomings by its own creator, Ross Ihaka, who has openly proposed to scrap the language altogether and start afresh by using a Lisp-based engine as the foundation for a statistical computing system [34, 53].

As a community repository of bioinformatics packages, BioLisp does not yet exist as such (albeit its name currently denotes the native language of BioBike [4, 54], a large-scale bioinformatics Lisp application), which means that there is certainly wide scope and potential for its rise and development in the bioinformatics community.

## Macros and domain-specific languages

Lisp is a so-called homoiconic language, which means that Lisp code is represented as a data structure of the language itself in such a way that its syntactical structure is preserved. In more technical terms, while the Lisp compiler has to parse the textual representation of the program (the ‘source code’) into a so-called abstract syntax tree (like any other compiler of any programming language has to), a Lisp program has direct access to (and can modify) this abstract syntax tree, which is presented to the program in a convenient, structured way.

This property enables Lisp to have a macro system that remains undisputed in the programming language world [55]. Although ‘macros’ in languages like C have the same name, they are essentially just text substitutions performed on the source code before it is compiled and they cannot always reliably preserve the lexical structure of the code. Lisp macros, on the other hand, operate at the syntactic level. They transform the program structure itself and, as opposed to C macros, are written in the same language they work on and have the full language available all the time. Lisp macros are thus not only used for moderately simple ‘find and replace’ chores but can apply extensive structural changes to a program. This includes tasks that are impossible in other languages. Examples would be the introduction of new control structures (while Python users had to wait for the language designers to introduce the ‘with’ statement in version 2.5, Lisp programmers could always add something like that to the language themselves), pattern matching capabilities (while Lisp does not have pattern matching like ML or Haskell out of the box, it is easy to add [56]) or the integration of code with markup languages (if you want you can, e.g., write code that mimics the structure of an HTML document it is supposed to emit [57, 58]).

In addition to that, Common Lisp even offers access to its ‘reader’, which means that code can be manipulated (in Lisp) before it is parsed [59]. This enables Lisp programs to completely change their surface syntax if necessary. Examples would be code that adds Perl-like interpolation capabilities to Lisp strings [60] or a library [61] that enables Lisp to read arithmetic in ‘infix’ notation, i.e. to understand ‘20 + 2 * 21’ in addition to the usual ‘(+ 20 (* 2 21))’.

These features make Lisp an ideal tool for the creation of domain-specific languages: languages that are custom-tailored to a specific problem domain but can still have access to all of Lisp. A striking example is Common Prolog [62], a professional Prolog system implemented and embedded in Common Lisp. In bioinformatics, the Biolingua [5] project (now called BioBike) built a cloud-based general symbolic biocomputing domain-specific language (DSL) entirely in Common Lisp. The system, which could be programmed entirely through the browser, was its own complete biocomputing language, which included a built-in deductive reasoner, called BioDeducta [54]. Biolingua programs, guided by the reasoner, would invisibly call tools such as BLAST [63] and Bioconductor [44] on the server-side, as needed. Symbolic biocomputing has also previously been used to create user-friendly visual tools for interactive data analysis and exploration [64].

## Other unique strengths

In addition to homoiconicity, Lisp has several other features that set it apart from mainstream languages:

- In Lisp, programmers usually work in a special incremental interactive programming environment called the read-eval-print loop (REPL) [65, 66]. This means that the Lisp system continuously reads expressions typed by the user, evaluates them and prints the results. The REPL enables a paradigm that allows the programmer to continually interact with their program as it is developed. This is similar to the way Smalltalk ‘images’ evolve [59] and different from the usual edit-compile-link-execute cycle of C-like languages. This approach lends itself well to explorative programming and rapid prototyping. As such, the REPL enables the programmer to write a function, test it, change it, try a different approach, etc., while never having to stop for any lengthy compilation cycles [24].
- Common Lisp was designed from the ground up to create large, complex and long-running applications and thus supports software ‘hot swapping’: the code of a running program can be changed without the need to interrupt it. This includes features like the ability of the Common Lisp object system (CLOS) to change the classes of existing objects. Although Erlang and Smalltalk also support hot swapping, no mainstream compiled language does this to our knowledge. Hot swapping can be performed in Java to a certain extent, but only with the help of third-party frameworks, as it is not an intrinsic feature of the language itself.
- Lisp invented exception handling, and Common Lisp, in particular, has an error-handling facility (the ‘condition system’ [24]) that goes far beyond most other languages: it does not necessarily unwind the stack if an exception occurs and instead offers so-called restarts to programmatically continue ‘where the error happened’. This system makes it easy to write robust software, which is an essential ingredient to building industry-strength fault-tolerant systems capable of handling a variety of conditions, a trait especially useful for artificial intelligence and machine learning applications. In the Bioconductor community, error-handling facilities are ubiquitously present in practically all R/Bioconductor packages via tryCatch(), a base R function whose roots originate directly from Lisp’s condition system.
- Common Lisp implementations usually come with a sophisticated ‘foreign function interface’ (FFI) [24], which allows direct access from Lisp to code written in C or C ++ and sometimes also to Java code. This enables Lisp programmers to make use of libraries written in other languages, making those libraries a direct strength of Lisp. For instance, it is simple to call Bioconductor from Lisp, just as Python and other programming languages can [67, 68]. Likewise, Clojure runs on the JVM and, thus, has immediate access to all of Java’s libraries.

It has been shown that these features, together with other amenities like powerful debugging tools that Lisp programmers take for granted, offer a significant productivity boost to programmers [3]. Lisp also gives programmers the ability to implement complex data operations and mathematical constructs in an expressive and natural idiom [69]. 

## Speed considerations

The interactivity and flexibility of Lisp languages are something that can usually only be found (if at all) in interpreted languages. This might be the origin of the old myth that Lisp is interpreted and must thus be slow—however, this is not true. Compilers for Lisp have existed since 1959, and all major Common Lisp implementations nowadays can compile directly to machine code, which is often on par with C code [70,71,72] or only slightly slower. Some also offer an interpreter in addition to the compiler, but examples like Clozure Common Lisp demonstrate that a programmer can have a compiler-only Common Lisp. For example, CL-PPCRE, a regular expression library written in Common Lisp, runs faster than Perl’s regular expression engine on some benchmarks, even though Perl’s engine is written in highly tuned C [24].

Although programmers who use interpreted languages like Python or Perl for their convenience and flexibility will have to resort to writing in C/C ++ for time-critical portions of their code, Lisp programmers can usually have their cake and eat it too. This was perhaps best shown with direct benchmarking by the creator of the R programming language, Ross Ihaka, who provided benchmarks demonstrating that Lisp’s optional type declaration and machine-code compiler allow for code that is 380 times faster than R and 150 times faster than Python [34]. And not only will the code created by Lisp compilers be efficient by default, Common Lisp, in particular, offers unique features to optimize those parts of the code (usually only a tiny fraction) that really need to be as fast as possible [59]. This includes so-called compiler macros, which can transform function calls into more efficient code at runtime, and a mandatory disassembler, which enables programmers to fine-tune time-critical functions until the compiled code matches their expectations. It should also be emphasized that while the C or Java compiler is ‘history’ once the compiled program is started, the Lisp compiler is always present and can thus generate new, fast code while the program is already running. This is rarely used in finished applications (except for some areas of AI), but it is an important feature during development and helpful for explorative programming.

To further debunk the popular misconception that Lisp languages are slow, Clojure was recently used to process and analyze SAM/BAM files [39] with significantly less lines of code and almost identical speeds as SAMTools [73], which is written in the C programming language. In addition, Common Lisp was recently used to build a high-performance tool for preparing sequence alignment/map files for variant calling in sequencing pipelines [6]. This HPC tool was shown to significantly outperform SAMTools and Picard on a variety of benchmarks [6].

## A case study: Pathway Tools

Pathway Tools [74, 75] is an example of a large bioinformatics software system written in Common Lisp (Allegro Common Lisp from Franz Inc.). Pathway Tools has among the largest functionality of any bioinformatics software system, including genome informatics, regulatory network informatics, metabolic pathway informatics and omics data analysis. For example, the software includes a genome browser that zooms from the nucleotide level to the chromosome level; it infers metabolic reconstructions from annotated genomes; it computes organism-specific layouts of metabolic map diagrams; it computes optimal routes within metabolic networks; and it can execute quantitative metabolic flux models.

The same Pathway Tools binary executable can execute as both a desktop window application and as a Web server. In Web server mode, Pathway Tools powers the BioCyc.org Web site, which contains 7600 organism-specific Pathway/Genome Databases, and services ∼500 000 unique visitors per year and up to 100 000 page views per day. Pathway Tools uses the ‘hot-swapping’ capabilities of Common Lisp to download and install software patches at user sites and within the running BioCyc Web server. Pathway Tools has been licensed by 7200 groups, and was found to have the best performance and documentation among multiple genome database warehousing systems [76].

Pathway Tools consists of 680 000 lines of Common Lisp code (roughly the equivalent of 1 400 000 lines of C or Java code), organized into 20 subsystems. In addition, 30 000 lines of JavaScript code are present within the Pathway Tools Web interface. We chose Common Lisp for development of Pathway Tools because of its excellent properties as a high-level, highly productive, easy-to-debug programming language; we strongly believe that the choice of Common Lisp has been a key factor behind our ability to develop and maintain this large and complex software system.

## A case study: BioBike

BioBike provides an example of a large-scale application of the power of homoiconicity. In personal communication, the inventor of BioBike, Jeff Shrager, explained why Lisp (in this case, Common Lisp) was chosen as the implementation language, an unusual choice even for the early 2000’s. According to Shrager, Lisp-style DSL creation is uniquely suited to ‘living’ domains, such as biology, where new concepts are being introduced on an ongoing basis (as opposed to, for example, electronics, where the domain is better understood, and so the conceptual space is more at rest). Shrager pointed out that as Lisp-based DSLs are usually implemented through macros, this provides the unique capability of creating new language constructs that are embedded in the home programming language (here, in Lisp). This is a critical distinction: in most programming languages, DSLs are whole new programming languages built on top of the base language, whereas in Lisp, DSLs are built directly into the language.

Lisp-based DSLs commonly show up in two sorts of domain-specific control structures: WITH- … clauses and MAP- … clauses. By virtue of Lisp’s homoiconicity, such constructs can take code as arguments, and can thereby create code-local bindings, and do various specialized manipulation directly on the code itself, in accord with the semantics of the new construct. In non-homoiconic languages, users must do this either by creating new classes/objects, or through function calls or via an ugly hack commonly referred to as ‘Greenspun’s 10th rule’ [77], wherein users must first implement a quasi-LFL on top of the base language, and then implement the DSL in that quasi-LFL. Both the object-creation and function-call means of creating new constructs lead to encapsulation problems, often requiring ugly manipulations such as representing code as strings, passing code-conditionalizing arguments, and then having to either globalize them, or re-pass them throughout a large part of the codebase. The Lisp-like methods of embedding DSLs into the base language via macros, one can simply use, for example, a WITH-GENES or a MAP-GENES macro wrapper, and within these, all one need do is to write normal everyday Lisp code, and the wrapper, because it has access to and can modify the code that gets run, has no such firewalls, enabling a much more powerful sort of computation. This greatly simplifies the incremental creation and maintenance of the DSL, and it is for this reason, argues Shrager, that Lisp (and LFLs more generally) is well suited to biology. Being a science that is creating new concepts constantly, it is especially important to be able to flexibly add concepts to the DSL.

BioBike was created by a team led by Jeff Shrager and JP Massar, and later Jeff Elhai. Its core Web listener is almost 15 000 lines of Common Lisp code in 25 modules, and the entire BioBike system is nearly 400 000 lines of code in about 850 modules, including the Web listener, many specialized bioinformatics modules, a scratch-like visual programming language (built using a specialized LFL that compiles to JavaScript, because of Peter Siebel), a specialized bioinformatics-oriented frame system (because of Mike Travers) and many other smaller modules.

## Perspectives and outlook

Historically speaking, Lisp is the second oldest (second only to Fortran) programming language still in use and has influenced nearly every major programming language to date with its constructs [78]. For example, it may be surprising to learn that R is written atop of Scheme [79]. In fact, R borrows directly from its Lisp roots for creating embedded domain-specific languages within R’s core language set [80]. For instance, ggplot2 [81], dplyr [82] and plyr [83] are all examples of DSLs in R. This highlights the importance and relevance of Lisp as a programmable programming language, namely the ability to be user-extensible beyond the core language set. Given the wide spectrum of domains and subdomains in bioinformatics and computational biology research, it follows that similar applications tailored to genomics, proteomics, metabolomics or other research fields may also be developed as extensible macros in Common Lisp. By way of analogy, perhaps a genomics equivalent of ggplot2 or dplyr is in store in the not-so-distant future. Advice for when such pursuits are useful is readily available [84]. Perhaps even more importantly, it is imperative to take into the consideration the future of statistical computing [34], which will form the big data backbone of artificial intelligence and machine learning applications in bioinformatics.

## Conclusions

New programming language adoption in a scientific community is both a challenging and rewarding process. Here, we advocate for and propose a greater inclusion of the LFLs into large-scale bioinformatics research, outlining the benefits and opportunities of the adoption process. We provide historical perspective on the influence of language choice on research trends and community standards, and emphasize Lisp’s unparalleled support for homoiconicity, domain-specific languages, extensible macros and error handling, as well as their significance to future bioinformatics research. We forecast that the current state of Lisp research in bioinformatics and computational biology is highly conducive to a timely establishment of robust community standards and support centered around not only the development of bioinformatic domain-specific libraries but also the rise of highly customizable and efficient machine learning and AI applications written in languages like Common Lisp, Clojure and Scheme.

**Key Points**

- Lisp empowers programmers to write faster programs faster. An empirical study shows that when programmers tackle the same problems in Lisp, C/C ++ and Java, that the Lisp programs are smaller (and therefore easier to maintain), take less time to develop and run faster.
- The Lisp family of programming languages (Common Lisp, Scheme and Clojure) makes it easy to create extensible macros, which facilitate the creation of modularized extensions to help bioinformaticians easily create plug-ins for their software. This, in turn, paves the way for creating enterprise-level, fault-tolerant domain-specific languages in any research area or specialization.
- The current state of Lisp research in bioinformatics and computational biology is at a point where an official BioLisp community is likely to be established soon, especially considering the documented shortcomings of mainstream programming languages like R and Python when compared side by side with identical implementations in Lisp.

## Notes

**Bohdan B. Khomtchouk** is an NDSEG Fellow and PhD candidate in the Human Genetics and Genomics Graduate Program at the University of Miami Miller School of Medicine. His research interests include bioinformatics and computational biology applications in HPC, integrative multi-omics, artificial intelligence, machine learning, mathematical genetics, biostatistics, epigenetics, visualization, search engines and databases.

## Notes

**Edmund Weitz** is full professor at the University of Applied Sciences in Hamburg, Germany. He is a mathematician and his research interests include set theory, logic and combinatorics.

## Notes

**Peter D. Karp** is the director of the Bioinformatics Research Group within the Artificial Intelligence Center at SRI International. Dr Karp has authored >130 publications in bioinformatics and computer science in areas including metabolic pathway bioinformatics, computational genomics, scientific visualization and scientific databases.

## Notes

**Claes Wahlestedt** is Leonard M. Miller Professor at the University of Miami Miller School of Medicine and is working on a range of basic science and translational efforts in his roles as Associate Dean and Center Director for Therapeutic Innovation. The author of some 250 peer-reviewed scientific publications, his ongoing research projects concern bioinformatics, epigenetics, genomics and drug/biomarker discovery across several therapeutic areas. He has experience not only from academia but also from leadership positions in the pharmaceutical and biotechnology industry.
