# How the strengths of Lisp-family languages facilitate building complex and flexible bioinformatics applications

**DOI:** 10.1093/bib/bbx016

**Published:** 2017-03-01

**Authors:** Bohdan B Khomtchouk, Edmund Weitz, Peter D Karp, Claes Wahlestedt

Briefings in Bioinformatics (2016) doi: 10.1093/bib/bbw130

In the above article, various author names were incorrectly abbreviated in the ‘References’ section.

The corrections have been made online and in print. The publisher apologizes for this error.

